# Kinetic Changes of Viremia and Viral Antigens of Hepatitis B Virus During and After Pregnancy

**DOI:** 10.1097/MD.0000000000002001

**Published:** 2015-11-13

**Authors:** Jingli Liu, Yongchun Bi, Chenyu Xu, Lanhua Liu, Biao Xu, Tingmei Chen, Jie Chen, Mingjie Pan, Yali Hu, Yi-Hua Zhou

**Affiliations:** From the Department of Experimental Medicine (JL, YB, MP, Y-HZ), Jiangsu Key Laboratory for Molecular Medicine (JL, MP, YH, Y-HZ); Department of Obstetrics and Gynecology (JC, YH); Department of Infectious Diseases, Nanjing Drum Tower Hospital, Nanjing University Medical School, Jiangsu, China (Y-HZ); Zhenjiang Fourth People's Hospital (CX, TC); and Department of Obstetrics and Gynecology, Taixing People's Hospital, Jiangsu, China (LL, BX).

## Abstract

Whether pregnancy may influence the replication of hepatitis B virus (HBV) remains unknown. The authors aimed to clarify this issue by observing the kinetics of HBV deoxyribonucleic acid (DNA) and viral antigens in women during and after pregnancy.

Total, 371 pregnant women with positive hepatitis B surface antigen (HBsAg) were enrolled. Serial sera collected during and after pregnancy were quantitatively measured for HBV DNA, HBsAg, and hepatitis B e antigen (HBeAg).

Total, 34 HBeAg-positive women underwent alanine aminotransferase (ALT) elevation during or after pregnancy; levels of HBV DNA and HBsAg in them showed no obvious change between second trimester or delivery and 7 to 12 months postpartum (*P* > 0.05). The 337 others had normal alanine aminotransferase levels during pregnancy and postpartum. In 147 HBeAg-positive women with follow-up 7 to 12 months postpartum, the average levels of HBV DNA (>7.0 log_10_ IU/mL), HBsAg (>4.0 log_10_ IU/mL), and HBeAg (>3.0 log_10_ S/CO) were longitudinally constant during pregnancy and postpartum, respectively. In 173 women with follow-up 4.8 years postpartum, neither HBV DNA levels nor antigen titers showed significant difference between second trimester and 4.8 years postpartum, regardless of the HBeAg status. In addition, levels of HBV DNA and viral antigens in second trimester, around delivery, 6 to 8 weeks and 7 to 12 months postpartum showed no marked fluctuations, respectively.

Serum levels of HBV DNA and viral antigens in HBsAg-positive women are highly constant during pregnancy and postpartum, regardless of the HBeAg status and alanine aminotransferase levels. This demonstrates that pregnancy has little influence on the HBV replication and antigen expression.

## INTRODUCTION

Hepatitis B virus (HBV) infection is a major cause of liver cirrhosis and hepatocellular carcinoma (HCC) worldwide. Globally, 350 million patients are chronically infected with HBV.^1^ In regions highly endemic for HBV infection, the infection is usually acquired via mother-to-infant transmission. In China, the hepatitis B surface antigen (HBsAg) carrier rate in pregnant women ranges from 6.0% to 7.8%.^2–6^ This prevalence is of particular concern as mother-to-infant transmission remains the predominant mode of HBV infection. The risk of perinatal transmission of HBV is associated with the HBV DNA levels and HBeAg status of mother, thus, it is important to investigate the influence of pregnancy on the viral replication and antigens expression in pregnant women infected with HBV. Little, however, is known whether pregnancy influences viremia levels in women with chronic HBV infection.

Hepatitis B virus is a noncytopathic virus and the associated hepatic inflammation is mediated by the host's immune responses. During pregnancy, several alterations in the maternal immune system allow mothers to tolerate the foetus,^7^ such as maternal alloreactive T-lymphocyte depletion, alloreactive Th1 cell blocking, increasing of the regulatory T-cells, and increasing apoptosis of activated maternal lymphocytes.^8,9^ The maternal immunosuppressive status may result in increased viremia levels or reactivation of latent virus, such as hepatitis C virus^10^ and cytomegalovirus.^11^ Whether the maternal immunosuppressive status during pregnancy may increase HBV replication is less studied, with inconsistent results.^12–14^ To clarify this issue, we therefore, measured serum HBV DNA concentrations and viral antigen titers in the serial samples from the HBsAg positive women during pregnancy and postpartum.

## METHODS

### Patients

During September 2008 to March 2014, a total of 371 HBsAg-positive pregnant women with normal alanine aminotransferase (ALT) levels before pregnancy were enrolled. Of them, 236 were HBeAg-positive, and no one had antiviral therapy history and coinfection with hepatitis C virus or human immunodeficiency virus. Serum samples were collected at the second trimester (gestational weeks 25–28), delivery (2 days before or after delivery), 6 to 8 weeks postpartum, 7 to 12 months postpartum, and long-term follow-up period (average 4.8 ± 1.1 years after labor), respectively. Each woman had at least 2 longitudinal samples. This study was approved by the institutional review boards of Nanjing Drum Tower Hospital, Taixing People's Hospital, and Zhenjiang Fourth People's Hospital. Each patient signed the informed consent.

### Detection of Hepatitis B Virus Markers

All serum samples were tested by commercial ELISA kits (Kehua Biotech, Shanghai, China) for the presence of HBsAg, anti-HBs, HBeAg, anti-HBe, and anti-HBc. Hepatitis B surface antigen and HBeAg were quantitatively tested with microparticle enzyme immunoassay (ARCHITECT, Abbott, North Chicago, USA); when the HBsAg levels were higher than 250 IU/mL, the sera were diluted to 1:20 to 1:1000 to obtain a reading within the range of the calibration curve. Hepatitis B virus DNA levels were quantitatively measured using real-time polymerase chain reaction (PCR) assay (Shenyou Biotech, Shanghai, China), with the detection limit 100 IU/mL.

### Hepatitis B Virus Genotyping

Serum DNA extracted from 200 μL of each serum using phenol/chloroform method was used for HBV genotyping, which was performed by nested polymerase chain reaction as previously described.^15^ The external primers for the PCR were C1, 5′-YTGGCCAAAATTCGCAGTC-3′ (nt 300–318), and C2, 5′-AAACCCCARRAGACCCACAA-3′ (nt 998–1017), and the internal primers were C3, 5′-CTCCARTCACTCACCAAC-3′, (nt 325–342), and C4, 5′-TGACAKACYTTCCAATCAAT-3′, (nt 973–992). The purified PCR products were subjected to DNA sequence analysis of the S region of HBV genome by ABI BigDye Terminator v3.1 sequencing kits (Applied Biosystems) and an ABI 3130 Genetic Analyzer (Applied Biosystems). All sequences were compared with different genotypes of HBV standard strains using Lasergene software, and the HBV genotyping was determined by phylogenetic analysis.

### Statistical Analysis

Statistical analysis was performed with the SPSS software (SPSS Standard version 19.0, SPSS Inc., Chicago, IL). The levels of HBsAg, HBeAg, and HBV DNA were expressed by the logarithm of measured values. Continuous variables normally distributed were expressed as mean ± standard deviation and compared by *t*-test between 2 groups or repeated measures analysis of variance. Quantitative data non-normally distributed were presented as median and range, and compared Bartlett test of sphericity. *P* < 0.05 was considered statistically significant.

## RESULTS

### Participant Characteristics

A total of 371 HBsAg-positive pregnant women with normal ALT levels before pregnancy were enrolled, and 226 (60.9%) were HBeAg-positive. The average age was 25.7 ± 3.4 years (21–34). Of the 371 women, 34 (9.16%) underwent ALT elevation during or after pregnancy, while 337 other women showed normal ALT levels throughout the study period. All 34 women with elevated ALT were HBeAg positive.

### Viral Replication and Antigens Expression in 34 Pregnant Women with Elevated Alanine Aminotransferase Levels in Pregnancy or Postpartum

Elevation of ALT in patients infected with HBV usually indicates the activation of immune responses against HBV, which may influence the viral levels in the circulation. Thus, we investigated the dynamic changes of HBV DNA and viral antigens in the circulation of pregnant women with increased and normal levels of ALT, respectively.

Totally, 34 women with HBeAg-positive had elevated ALT levels. Hepatitis B virus genotypes were B in 15 (46.9%), C in 17 (53.1%), and unclassified in 2 women, respectively. Total, 19 women with normal ALT levels during pregnancy had increased ALT levels, 7 to 12 months after delivery (Table 1). The comparison of the levels of HBV DNA, HBsAg, and HBeAg between the second trimester of delivery and 7 to 12 months postpartum are presented in Table 1. There were no obvious changes in HBV DNA levels and HBsAg titers, while HBeAg titers decreased slightly 7 to 12 months after delivery (*P* = 0.025 and 0.034, respectively). There were no significant differences in HBV DNA levels and antigen titers between second trimester and during delivery, although the pregnant women were not same subjects.

**TABLE 1:**
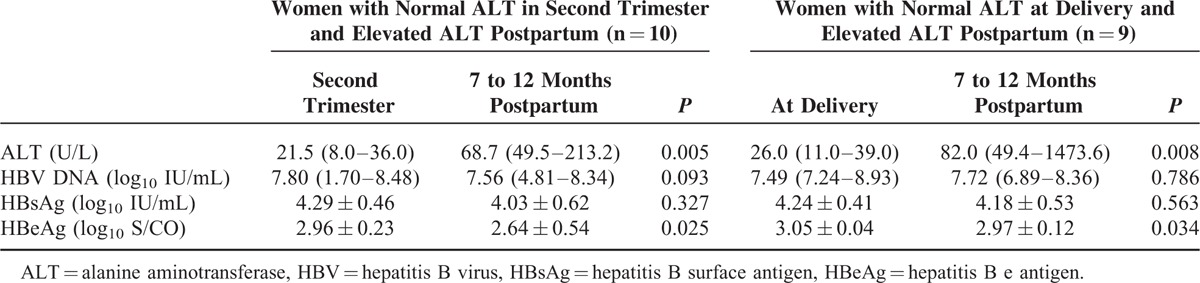
Levels of Hepatitis B Virus DNA and Viral Antigens During Pregnancy and After Delivery in Women with Elevated Alanine Aminotransferase Postpartum

Of the 15 pregnant women with elevated ALT during pregnancy, 9 presented normal ALT and 6 others still had elevated levels 7 to 12 months after delivery. As shown in Table 2, serum levels of HBV DNA and viral antigens showed no significant change in pregnancy and postpartum periods, regardless of the ALT levels.

**TABLE 2:**
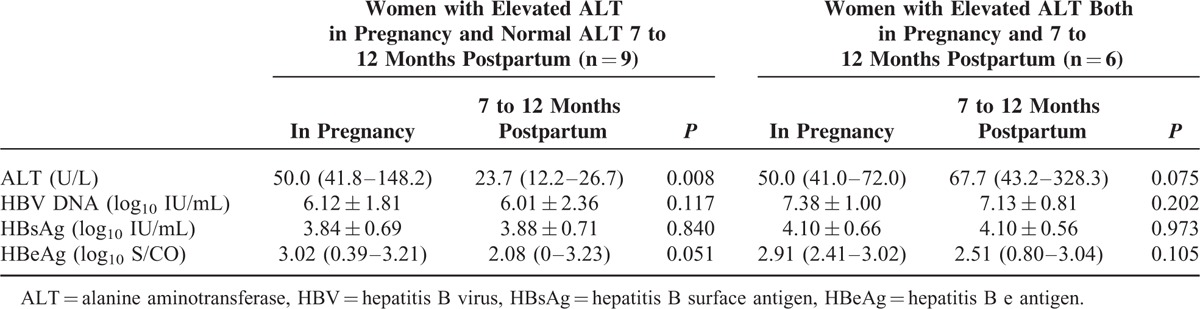
Levels of Hepatitis B Virus DNA and Viral Antigens During Pregnancy and After Delivery in Women with elevated Alanine Aminotransferase During Pregnancy

### Dynamic Change of Hepatitis B Virus DNA and Viral Antigens in 337 Women with Normal Alanine Aminotransferase Levels

A total of 337 (90.8%) pregnant women with positive HBsAg maintained normal ALT levels before and during pregnancy and 7 to 12 months postpartum; 192 were also HBeAg positive and 145 others were HBeAg negative. Of the 192 HBeAg-positive women, serial serum samples were collected from the second trimester (77 sera) and at delivery (70 samples), respectively; all these 147 women were followed-up 7 to 12 months postpartum. Hepatitis B virus genotypes were B in 54 (37.5%), C in 90 (62.5%), and undetermined in 3 women, respectively. As shown in Table 3, neither HBV DNA levels nor viral antigens titers displayed significant fluctuation from pregnancy to 7 to 12 months postpartum. In addition, serum levels of HBV DNA and viral antigens were comparable between 77 women in second trimester and 70 women at delivery.

**TABLE 3:**
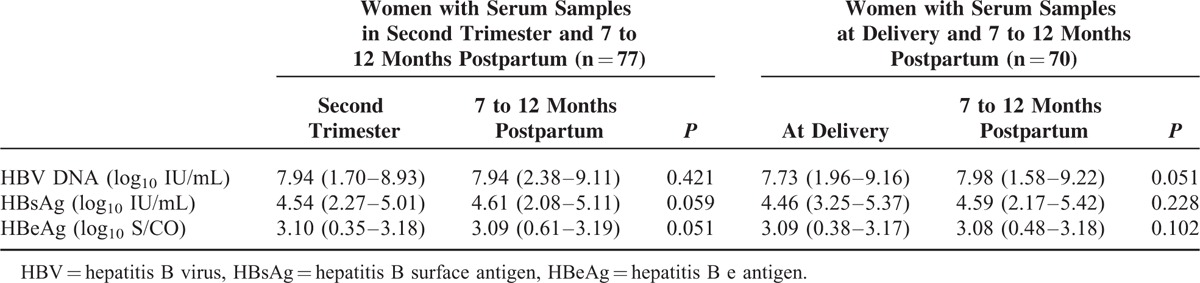
Levels of Hepatitis B Virus DNA and Viral Antigens During Pregnancy and 7 to 12 Months Postpartumin 147 Women with Normal Alanine Aminotransferase

Of the 337 women with normal ALT levels, 173 were followed up 4.8 ± 1.1 years postpartum. Of them, 28 were HBeAg-positive, and HBV genotypes were B in 5 (17.86%) and C in 23 (82.14%) women, respectively, and 145 others were HBeAg-negative, and HBV genotypes were B in 45 (31.25%), C in 99 (68.75%), and undetermined in 1 women, respectively. As shown in Table 4, neither HBV DNA levels nor antigens titers showed significant difference between second trimester and 4.8 years postpartum, regardless of the HBeAg status. In addition, the median HBV DNA levels in HBeAg-positive women were > 5 log_10_ IU/mL higher than those in HBeAg-negative women.

**TABLE 4:**
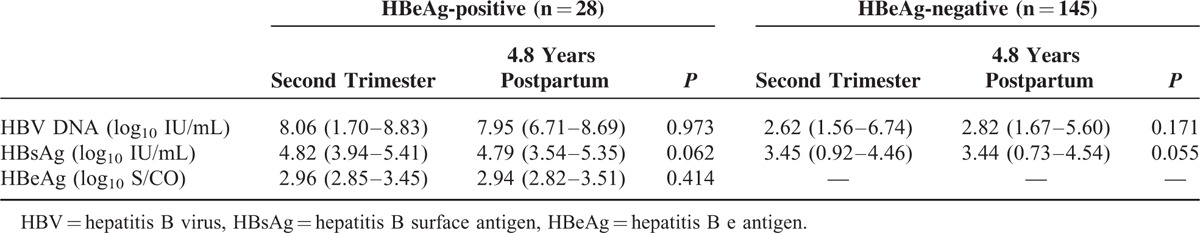
Levels of Hepatitis B Virus DNA and Viral Antigens During Pregnancy and Long-Term Postpartumin 173 Women with Normal Alanine Aminotransferase

**FIGURE 1:**
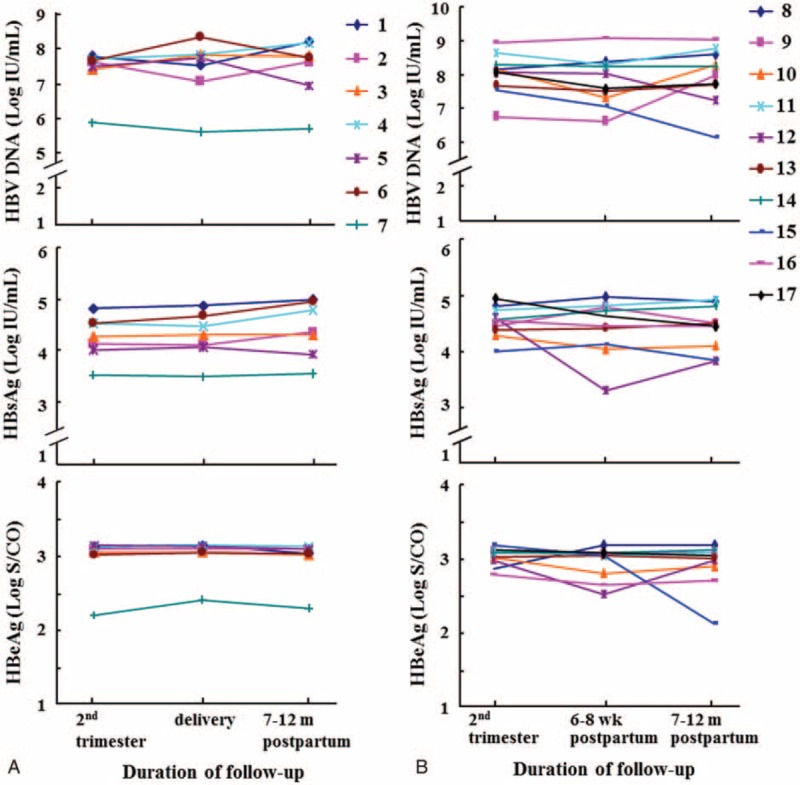
Dynamic change of hepatitis B virus DNA and viral antigens in second trimester, around delivery, 6 to 8 weeks and 7 to 12 months postpartum. Each number denotes an individual woman. A, During second trimester, delivery and 7 to 12 months postpartum, respectively (n = 7). B, During second trimester, 6 to 8 weeks and 7 to 12 months postpartum, respectively (n = 10).

Of the 337 women, 17 HBeAg-positive women with 3 serial serum samples were observed to detect the viral dynamic changes. Hepatitis B virus genotypes were B in 8 (47.1%) and C in 9 (52.9%), respectively. Of them, 7 patients were followed up in second trimester, just before delivery, and 7 to 12 months postpartum, and 10 others were followed up in second trimester, 6 to 8 weeks and 7 to 12 months postpartum, respectively. Figure 1 shows the serum levels of HBV DNA, HBsAg, and HBeAg over time in these women, respectively. The results revealed no obvious change of HBV DNA levels in each of the 17 women, with slight fluctuation by less than 1 log_10_ IU/mL. Similarly, none of them showed significant dynamic difference in HBsAg and HBeAg titers over the time.

## DISCUSSION

In the current study, we investigated the dynamic change of HBV DNA and viral antigens in the circulation of HBsAg-positive women during and after pregnancy. Our results revealed that the levels of HBV DNA and the antigens were highly constant during pregnancy and postpartum, regardless of the HBeAg status and ALT levels. This indicates that pregnancy has little influence on the HBV replication and antigens expression.

In the current study, although we did not compare the virologic parameters of these women before and during pregnancy and after delivery, because of the difficulty to collect serum samples before pregnancy, we consider that our conclusion is supported by the data. First, we enrolled a large panel of pregnant women with HBV infection, which may minimize the bias caused by sampling. Second, we compared the virologic parameters of pregnant women in midterm, around delivery, 7 to 12 months and 4.8 years postpartum, respectively. Third, the duration of maternal immune suppression in pregnancy is estimated to be not more than 2 to 3 months after delivery,^16^ and the maternal immunosuppressive status during the pregnancy should be disappeared in 7 to 12 months and 2 to 5 years postpartum; however, there was no obvious decrease in HBV DNA levels or titers of HBsAg during pregnancy and 7 to 12 months and 4.8 years postpartum. Therefore, the current investigation validates that pregnancy has little influence on the replications of HBV.

As previously reported,^5,17^ HBV DNA in the circulation showed a mean difference of 5 to 6 log_10_ IU/mL between HBeAg-positive and HBeAg-negative women in the current study. Regardless of the HBeAg status, our data showed that HBV DNA levels during pregnancy and postpartum were constant (Tables 3 and 4). Because individuals with normal ALT levels, positive HBeAg and high levels of HBV DNA are in the immunotolerant phase,^18^ one may assume that the maternal immunosuppression during pregnancy may have little influence on the replication of HBV in an “already existing” immune tolerant status. Individuals with normal ALT levels, positive anti-HBe and low levels of HBV DNA, however, are considered to be in the inactive HBV carrier status.^18^ If the maternal immunosuppression during pregnancy may activate the replication of HBV, the HBV DNA levels in pregnant women in the inactive HBV carrier status should have a trend to increase. Such a trend, however, was not observed in the current study.

It is reported that abnormal liver functions is often detected in pregnant women with HBV infection.^12,19^ In our study, of the 371 women, 15 (4.0%) experienced ALT elevation during pregnancy, 9 of whom had normal ALT levels after delivery. This indicates that the exaggeration of liver functions may be associated with the increased burden of liver because of pregnancy. On the contrary, 19 (5.1%) pregnant women had elevated ALT after delivery. At the time, it is difficult to distinguish whether the ALT elevation was caused by the T cell-mediated HBV-specific immune response^20^ or caused by overburden of taking care of the infants. Nevertheless, since HBV DNA levels in these women were longitudinally constant before and after elevation of ALT, the occurrence of abnormal liver functions is less likely associated with reactivation of HBV replications.

It is worth noting that in 19 patients with elevated ALT levels 7 to 12 months after delivery, the HBeAg titers decreased slightly postpartum, compared with that during pregnancy (Table 1). This may be in part because these mothers were already in the immunoclearance phase, but not related to pregnancy, since that most of pregnant women with positive HBeAg did not show the decrease of HBeAg levels postpartum (Tables 3 and 4, Figure 1). However, the HBV DNA levels in these 19 patients remained stable, indicating that the immunoclearance was not enough to clear the virus.

A major limitation in our study is that we did not measure the detailed prepregnancy virologic parameters, and could not compare the levels of HBV DNA and viral antigens between, before, and during pregnancy, because of the difficulty to collect the serum samples. Based on the constant levels of HBV DNA and viral antigens during mid-term and late pregnancies and 7 to 12 months and 2 to 5 years postpartum, however, it was very likely that the levels of HBV DNA and viral antigens before pregnancy had no remarkable difference. Thus, the lack of virologic parameters before pregnancy should not impede the conclusion that pregnancy may not activate HBV replication.

In summary, serum levels of HBV DNA and viral antigens in HBsAg-positive women were highly constant during pregnancy and postpartum, regardless of the HBeAg status and ALT levels. This demonstrates that pregnancy has little influence on the HBV replication and antigen expression.
